# Impact of age on the cerebrospinal fluid spaces: high-convexity and medial subarachnoid spaces decrease with age

**DOI:** 10.1186/s12987-022-00381-5

**Published:** 2022-10-28

**Authors:** Yosuke Hidaka, Mamoru Hashimoto, Takashi Suehiro, Ryuji Fukuhara, Tomohisa Ishikawa, Naoko Tsunoda, Asuka Koyama, Kazuki Honda, Yusuke Miyagawa, Kazuhiro Yoshiura, Shuken Boku, Kazunari Ishii, Manabu Ikeda, Minoru Takebayashi

**Affiliations:** 1 Department of Neuropsychiatry, Kumamoto University Hospital, Kumamoto, Japan; 2 Department of Neuropsychiatry, Faculty of Medicine, Kindai University, Osaka, Japan; 3 Department of Psychiatry, Osaka University Graduate School of Medicine, Osaka, Japan; 4 Department of Psychiatry, Kagoshima University Graduate School of Medical and Dental Sciences, Kagoshima, Japan; 5 Department of Psychiatry, Arao Kokoronosato Hospital, Kumamoto, Japan; 6 Department of Neuropsychiatry, Faculty of Life Sciences, Kumamoto University, Kumamoto, Japan; 7 Department of Geriatric Psychiatry, Mitsugumachi Clinic, Kumamoto, Japan; 8 Department of Rehabilitation and Physical Medicine, Kagoshima University Graduate School of Medical and Dental Sciences, Kagoshima, Japan; 9 Department of Radiology, Faculty of Medicine, Kindai University, Osaka, Japan

**Keywords:** Aging, Brain morphology, Cognitive dysfunction, Cerebrospinal fluid dynamics, Disproportionately enlarged subarachnoid-space hydrocephalus, Idiopathic normal-pressure hydrocephalus

## Abstract

**Background:**

Impaired cerebrospinal fluid (CSF) dynamics may contribute to the pathophysiology of neurodegenerative diseases, and play a crucial role in brain health in older people; nonetheless, such age-related changes have not been well elucidated. Disproportionately enlarged subarachnoid-space hydrocephalus (DESH) is a neuroimaging phenotype of idiopathic normal-pressure hydrocephalus, originating from impaired CSF dynamics, and closely associated with aging. This study aimed to investigate the pathophysiology of DESH and determine age-related changes in CSF dynamics.

**Methods:**

Using magnetic resonance imaging, we investigated the pathophysiology of DESH by quantitatively evaluating the volumes of DESH-related regions (ventricles [VS], Sylvian fissure [SF], and subarachnoid spaces at high convexity and midline [SHM]) and brain parenchyma in community-dwelling individuals aged  ≥ 65 years. DESH-related regions were assessed using a visual rating scale, and volumes measured using voxel-based morphometry. Brain parenchyma volumes were measured using FreeSurfer software.

**Results:**

Data from 1,356 individuals were analyzed, and 25 (1.8%) individuals had DESH. Regarding the relationships between the volume of each CSF space and age, VS and SF volumes increased with age, whereas SHM volume did not increase. VS and SF volumes increased as the whole brain volume decreased, whereas SHM volume did not increase even if the whole brain volume decreased; that is, SHM did not expand even if brain atrophy progressed. Moreover, lower Mini-Mental State Examination scores were significantly associated with lower SHM volume and higher VS volume. These associations remained significant even when individuals with DESH were excluded.

**Conclusions:**

This study showed that the volume of high-convexity and medial subarachnoid spaces did not expand and tended to decrease with age; the human brain continuously progresses toward a “DESH-like” morphology with aging in community-dwelling older persons (i.e., DESH might be an “accelerated aging stage” rather than an “age-related disorder”). Our results indicated that brain atrophy may be associated with the development of “DESH-like” morphology. In addition, this morphological change, as well as brain atrophy, is an important condition associated with cognitive decline in older adults. Our findings highlight the importance of investigating the aging process of CSF dynamics in the human brain to preserve brain health in older people.

**Supplementary Information:**

The online version contains supplementary material available at 10.1186/s12987-022-00381-5.

## Background

Dementia is a serious social problem worldwide, and suitable measures for its prevention and treatment are urgently needed. Age is the greatest risk factor for the development of dementia, and age-related pathological changes in the brain parenchyma are well studied [1]. Neuropathological changes, such as neurofibrillary tangles, neuritic plaques, and infarcts, increase with age, thereby reducing the brain volume, resulting in cognitive decline. Recently, various concepts of cerebrospinal fluid (CSF) dynamics as the clearance systems for protein waste such as amyloid β (e.g., the glymphatic system and the meningeal lymphatic system) have attracted attention [2–6]. Impaired CSF dynamics may contribute to the pathophysiology of neurodegenerative diseases such as Alzheimer’s disease [2, 5, 7–9]. CSF dynamics may have a crucial role in brain health; nonetheless, age-related changes in CSF dynamics have not been well elucidated.

Idiopathic normal-pressure hydrocephalus (iNPH) originates from impaired CSF dynamics in the absence of a previous illness or injury (e.g., meningitis and subarachnoid hemorrhage) [10]. Gait disturbance is the most prominent symptom, although cognitive impairment and urinary incontinence are also common. iNPH develops in older individuals, and shunt surgery improves symptoms [10]. Disproportionately enlarged subarachnoid-space hydrocephalus (DESH), is defined as tight high-convexity and medial subarachnoid spaces and enlarged Sylvian fissure (SF) with ventriculomegaly [11, 12], and is a neuroimaging phenotype of iNPH. DESH is closely associated with aging [13]. Thus, investigating the pathophysiology of DESH could reveal one aspect of age-related changes in CSF dynamics.

While the brain parenchyma volume and pathophysiology of CNS aging and neurological diseases have been extensively studied, few studies have quantified the volume of DESH-related regions (e.g., ventricles, SF, and high-convexity and medial subarachnoid spaces) in large-scale samples. One study reported the creation of an automated classifier for the imaging characteristics of DESH (DESH score) using the support vector machine-based method [14]; other studies investigated the association between the DESH score and cognitive dysfunction in community-dwelling individuals [15, 16]. High-convexity tight sulci (HCTS) have been reported using the DESH score, with 6.6% of community-dwelling older people having high-convexity tight sulci (HCTS) [15]. Individuals with HCTS tended to have cognitive dysfunction, often without Alzheimer’s disease pathology, which revealed a novel etiology called CSF dynamics disorders [15]. The DESH score is reportedly an independent predictor of subsequent cognitive decline in the general population [16]. These studies are valuable in terms of quantifying a DESH-like imaging pattern in large-scale samples of community-dwelling individuals and show that age-related changes in CSF dynamics may cause cognitive decline in older adults. However, it has not yet been established whether DESH is a rare condition associated with aging or whether the human brain continuously progresses toward a “DESH-like” morphology with aging (age-related disorder vs. accelerated aging stage, respectively). The DESH score did not represent the actual CSF volume of DESH-related regions; thus, the effect of brain atrophy (which should be closely associated with CSF space dilatation) on DESH remains unclear.

## Methods

### Aim

In this study, to investigate the pathophysiology of DESH, the volumes of DESH-related regions (ventricles, SF, and high-convexity and medial subarachnoid spaces) and brain parenchyma were quantitatively evaluated in a large sample of community-dwelling individuals aged  ≥ 65 years. Our objectives were (i) to examine whether DESH is an age-related disorder or an accelerated aging stage; (ii) to clarify whether brain atrophy is associated with the development of DESH-like morphology; (iii) to explore the factors associated with the development of DESH-like morphology; and (iv) to verify whether age-related DESH-like morphological changes are associated with clinical symptoms such as cognitive and gait dysfunctions.

### Study design and participants

A total of 1,577 community-dwelling older residents in Arao City, Kumamoto Prefecture (southern Japan), were enrolled between November 2016 and March 2017. This cross-sectional analysis using baseline data was part of the Japan Prospective Studies Collaboration for Aging and Dementia, which is designed to enroll approximately 10,000 community-dwelling residents aged  ≥ 65 years from eight sites in Japan to explore the genetic and environmental risk factors for dementia [17]. Participants were excluded if they had no or unsuitable magnetic resonance imaging (MRI) data, missing data, dementia (i.e., to examine the age-related changes in CSF dynamics), or severe gait disturbance (i.e., to exclude the effect of musculoskeletal disorders on gait assessment) (see Additional file 1: Table S1–S4).

### Standard protocol approvals, registrations, and patient consents

This study was approved by the Research Ethics Committee of Kumamoto University (Kumamoto, Japan; approval number, GENOME-333). All participants provided written informed consent prior to data collection in accordance with the Declaration of Helsinki.

## Procedures

Standardized approaches for questionnaires, blood tests, and dementia diagnosis were applied across all study sites, as previously described [17]. Cognitive function was assessed using the Mini-Mental State Examination (MMSE) [18]. Dementia [19] and its subtypes [17] were diagnosed based on standard criteria, and Petersen’s criteria were used to diagnose mild cognitive impairment (MCI) [20]. Individuals without dementia or MCI were considered cognitively normal in this study.

Gait was assessed using the Timed Up and Go (TUG) test [21]. The TUG test was performed twice, and the shorter time was used for the analysis. Gait disturbance was defined as a TUG time  > 12.0 s [22], and severe gait disturbance as a TUG time  > 16.7 s (three standard deviations above the mean of this cohort). Hypertension was defined as a blood pressure  ≥ 140/90 mmHg and/or the use of antihypertensive agents. Diabetes was defined as fasting blood glucose  ≥ 126 mg/dL, casual blood glucose  ≥ 200 mg/dL, hemoglobin A1c ≥ 6.5%, and/or the use of glucose-lowering agents. Dyslipidemia was defined as low-density lipoprotein  ≥ 140 mg/dL and/or high-density lipoprotein  < 40 mg/dL, and/or taking medication for dyslipidemia. Body mass index (BMI) was calculated using body height and weight. The criteria for atrial fibrillation were based on self-reported questions and/or electrocardiographic evidence. Information on education level, history of coronary artery disease, heart failure, and smoking was obtained using self-reported questionnaires.

### Imaging and diagnoses

Brain MRI was conducted at the Arao Municipal Hospital (Kumamoto, Japan) and Omuta Tenryo Hospital (Fukuoka, Japan) using the 1.5-Tesla Ingenia CX dual scanner (Philips Healthcare, Best, Netherlands) or the 1.5-Tesla Signa HDxt Ver.23 scanner (GE Healthcare, Milwaukee, WI, USA). The Philips MRI scanning protocol consisted of a three-dimensional (3D) T1-weighted sequence (repetition time = 8.6 ms, echo time = 4.0 ms, flip angle = 9°, matrix = 192 × 192, slice thickness = 1.2 mm), a 3D T2-weighted sequence (repetition time = 5082.3 ms, echo time = 100.0 ms, flip angle = 90°, matrix = 356 × 248, slice thickness = 5.0 mm), a 3D fluid-attenuated inversion recovery (FLAIR) sequence (repetition time = 11,000.0 ms, echo time = 120.0 ms, flip angle = 90°, matrix = 288 × 203, slice thickness = 5.0 mm), and a susceptibility-weighted imaging (SWI) sequence (repetition time = 78.4 ms, echo time = 41.4 ms, flip angle = 20°, matrix = 88 × 272, slice thickness = 2.4 mm). The GE Signa MRI scanning protocol consisted of a 3D T1-weighted sequence (repetition time = 8.3 ms, echo time = 3.4 ms, flip angle = 8°, matrix = 192 × 192, slice thickness = 1.2 mm), a 3D T2-weighted sequence (repetition time = 4517.0 ms, echo time = 92.6 ms, flip angle = 90°, matrix = 352 × 224, slice thickness = 5.0 mm), a 3D FLAIR sequence (repetition time = 10,000.0 ms, echo time = 149.2 ms, flip angle = 90°, matrix = 288 × 193, slice thickness = 5.0 mm), and a 3D T2-Star weighted angiography sequence (repetition time = 75.2 ms, echo time = 57.9 ms, flip angle = 20°, matrix = 320 × 200, slice thickness = 3.0 mm).

FLAIR MRI was used to assess vascular diseases such as infarction and white matter hyperintensity (WMH) load. Participants with large vascular lesions (e.g., cortical infarction and hemorrhage), tumors, and artifacts were excluded from the analysis (see Additional file 1: Table S3). The degree of WMH load was rated visually on axial FLAIR images by using the Fazekas scale (i.e., grade 1 [punctate], grade 2 [early confluent], or grade 3 [confluent]) in the periventricular and deep white matter (WM) regions [23]. The sum of the periventricular and deep WMH scores, ranging from 0 to 6, was used for the analysis. Lacunar infarction was defined as a CSF-like hypo-intensity with a diameter  > 2 mm surrounded by a rim of hyperintensity on T2 FLAIR. Lacunar infarction was considered present if there was at least one visible lacunar infarction. Microbleeds were assessed using SWI or T2-Star software and recorded using the same method used for lacunar infarction. The Fazekas scale, lacunar infarction, and microbleeds were the covariates. All brain images were assessed by a neuroradiologist (N.T.) and two neuropsychiatrists (Y.H. and M.H.) who were blinded to the clinical data. Each of the three evaluators separately assessed all brain images. In discordant cases, the rating was determined by the consensus of the three evaluators.

**Fig. 1:**
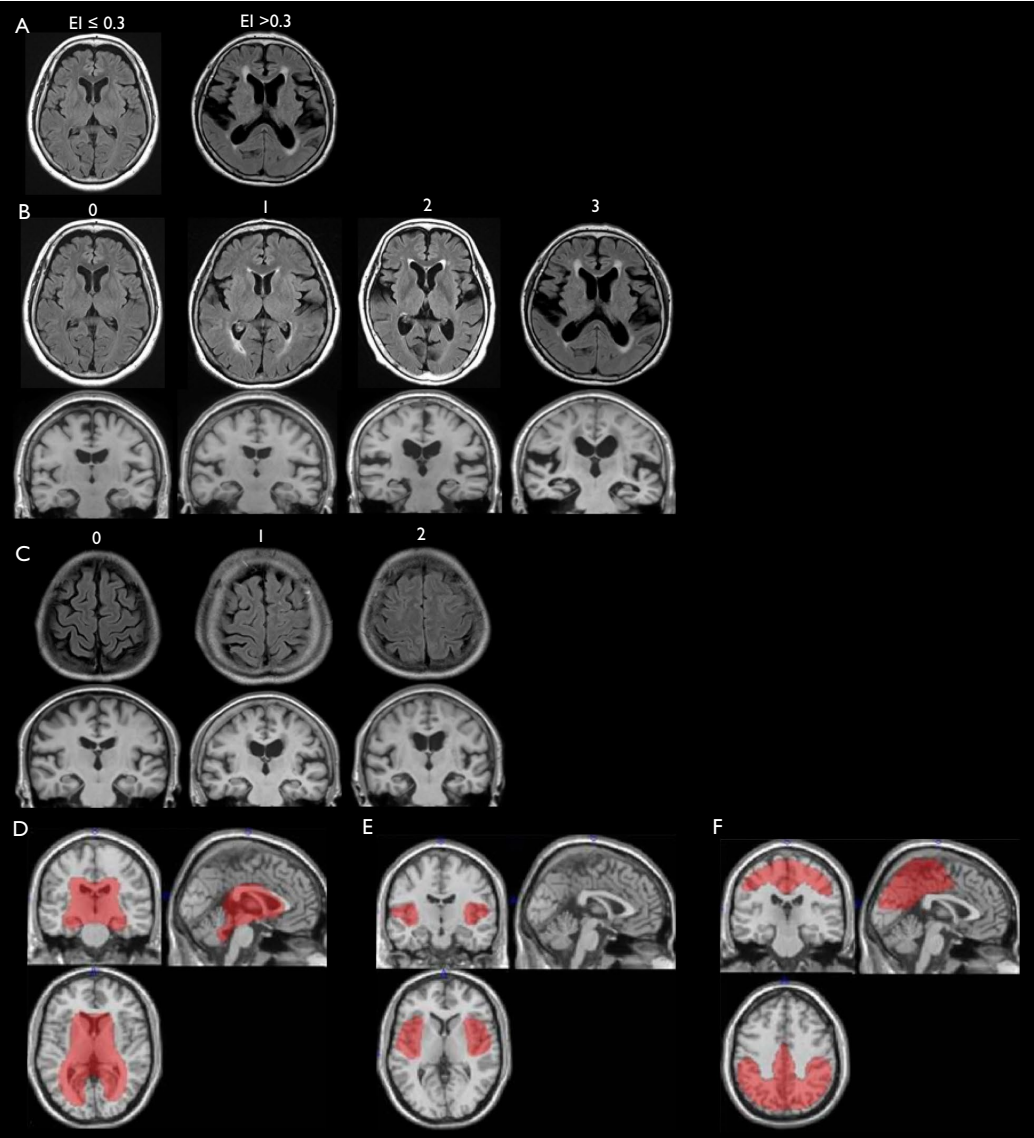
Assessment of DESH-related regions. **A** VS: EI ≤ 0.3, not dilated; EI > 0.3, dilated. Ventriculomegaly was defined as an EI of > 0.3. **B** SF: 0, normal; 1, mildly dilated; 2, moderately dilated; 3, severely dilated. Scores of 2 or 3 were defined as enlarged SF. **C** SHM: 0, not tight; 1, moderately tight; 2, severely tight. Scores of 1 or 2 were defined as tight SHM. **D** VOI template for the CSF volume of VS. **E** VOI template for the CSF volume of SF. **F** VOI template for the CSF volume of SHM. *DESH* disproportionately enlarged subarachnoid-space hydrocephalus, *EI* Evans index, *SF* Sylvian fissure, *SHM *subarachnoid space at high convexity and midline, *VOI *voxels of interest, *VS *ventricular system

A visual rating scale was used to assess the DESH-related regions with reference to the previous literatures [11, 12] (Fig. 1A–C). The Evans index (EI) value was the ratio of the maximum diameter of the frontal horns of the lateral ventricles to the maximum inner diameter of the skull on the transverse section. The ventricular system (VS) was classified as “not dilated” if EI ≤ 0.3 or “dilated” if EI > 0.3 (Fig. 1A). Ventriculomegaly was defined as an EI > 0.3. The SF was assessed in transverse and coronal sections. SF enlargement was rated as, 0 normal; 1, mildly dilated; 2, moderately dilated; and 3, severely dilated (Fig. 1B). Scores of 2 or 3 indicated enlarged SF. The subarachnoid spaces at high convexity and midline (SHM) were evaluated using transverse and coronal section images, and their tightness was rated as follows: 0, not tight; 1, moderately tight; and 2, severely tight (Fig. 1C). Scores of 1 or 2 constituted tight SHM.

Participants meeting all criteria for ventriculomegaly, enlarged SF, and tight SHM were diagnosed with DESH. “Possible iNPH with MRI support” was defined as DESH with MCI and/or gait disturbance. “Asymptomatic ventriculomegaly with features of iNPH on MRI (AVIM)” was defined as DESH with neither MCI nor gait disturbance.

To quantitatively assess the DESH-related regions, we utilized an automatic volumetric segmented brain image system, which was modified to evaluate iNPH [24, 25]. We prepared voxels of interest (VOI) templates for the intracranial volume, VS, SF, and SHM (Fig. 1D–F), as previously described [26]. Each regional VOI template was produced on a digital phantom Simulated Brain Database (http://www.bic.mni.mcgill.ca/brainweb/), according to the standard Montreal Neurological Institute space, with manual delineation of the contours of each structure. The SHM VOI template was produced manually by referring to the results of a previous study on voxel-based morphometry (VBM) in iNPH patients and normal controls [27]. For this process, the MRI for each participant was segmented into gray matter (GM), WM, and CSF by the SPM8 segmentation program (Wellcome Trust Centre for Neuroimaging, London, UK). The GM template image derived from the Simulated Brain Database was then spatially transformed into the GM image for each participant, and a normalization parameter was produced by using SPM8 and the Diffeomorphic Anatomical Registration Through Exponentiated Lie Algebra technique. This normalization parameter functions in the same manner as a reverse parameter produced in the anatomical normalization of an individual brain to a standard brain. With this parameter, the intracranial volume, VS, SF, and SHM VOI templates were transformed to each participant’s space. The intracranial volume was adjusted by using an image derived from the segmented GM, WM, and CSF images. The segmented images were derived by calculating the WM and GM areas with the voxels from the intracranial volume VOI template. CSF volumes of the VS, SF, and SHM were calculated using the transformed VS, SF, and SHM subarachnoid space VOI templates for each participant. Each regional volume was normalized to the total intracranial volume.

Volumetric segmentation was achieved using FreeSurfer version 5.3 (http://surfer.nmr.mgh.harvard.edu/) on CentOS6. Using the Desikan–Killany Atlas, we measured 34 cortical regions, 7 subcortical regions, corpus callosum, cerebellum cortex, and vessels (represented perivascular space around the basal ganglia) in absolute volumes (see Additional file 1: Table S5) [28, 29]. The vessel volume was used as a covariate. Each regional volume was normalized to the total intracranial volume.

### Statistical analysis

Statistical analysis was conducted using SPSS version 27.0 (IBM Corp., Armonk, NY, USA). To examine whether the volume of DESH-related regions showed continuity between normal aging and DESH, we analyzed the data in two groups: all individuals (*n* = 1,356) and the non-DESH group (*n* = 1,331).

Continuous variables were compared using t-tests. Differences in proportions were compared using the chi-square test. We used Pearson’s correlation coefficient (*r*) to determine the association between the EI and VS volume. Spearman rank correlation (*rs*) was used for comparing the visual rating scales of SF and SHM with their volumes. To determine the association between the VS, SF, and SHM volumes and age, we used Pearson’s correlation coefficient (*r*). To explore the factors associated with DESH-related regions and examine correlations between brain structure volumes and age, we used multivariate linear regression while adjusting for the following possible confounders: sex, education, hypertension, diabetes mellitus, dyslipidemia, atrial fibrillation, coronary artery disease, heart failure, BMI, history of smoking, Fazekas score, lacunar infarction, microbleeds, and perivascular space. We used Pearson’s correlation coefficient (*r*) to determine the association between the VS, SF, and SHM volumes and total brain volume.

To identify the brain structures, including DESH-related regions (i.e., VS, SF, SHM), that affect cognitive function, we conducted hierarchical multiple regression analysis using the MMSE score as the dependent variable. After entering the independent variables (age, sex, education, hypertension, diabetes mellitus, dyslipidemia, atrial fibrillation, coronary artery disease, heart failure, BMI, history of smoking, MRI scanner, Fazekas score, lacunar infarction, microbleeds, and perivascular space) in Block 1, each brain structure was entered in Block 2. For each analysis, the standardized regression coefficient (β_STD_) and the change in *R*^2^ (∆*R*^2^) were calculated. We ranked the effect of the brain structures on the MMSE score by descending ∆*R*^2^. The same analysis was conducted to evaluate the brain structures affecting gait function (i.e., TUG score).

For all multivariate analyses, we visually inspected normal Q-Q plot to check for normality of residuals. Collinearity was examined using the variance inflation factor (values  > 10 were considered problematic). The Durbin–Watson statistic was used to identify autocorrelation (values  < 1 and  > 3 were considered problematic). Cook’s distance was calculated to check for influential outliers (values  > 0.5 were considered problematic).

For all analyses, significance was set at *P* < 0.05. To correct for multiple comparisons, we employed Bonferroni corrections; for example, the significance level for the comparison of 46 brain structures (43 brain regions and three DESH-related regions) was *P* < 0.0011 (0.05/46).

## Results

**Fig. 2:**
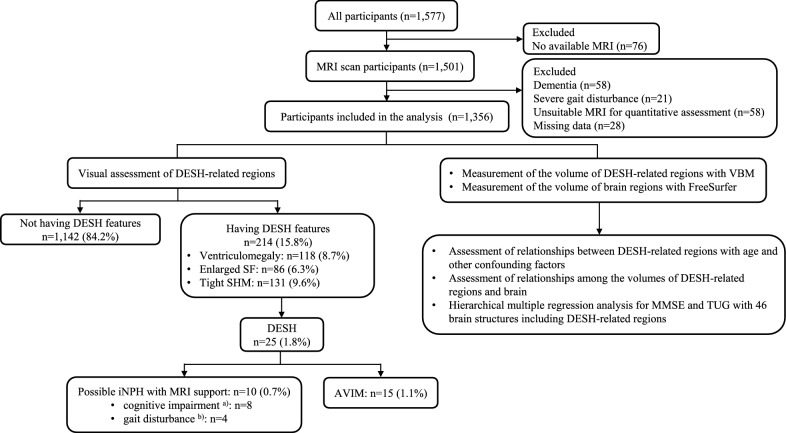
Flowchart of the study and the prevalence of DESH. ^a^MCI and ^b^TUG > 12.0 s. *AVIM *asymptomatic ventriculomegaly with features of idiopathic normal-pressure hydrocephalus on MRI, *DESH *disproportionately enlarged subarachnoid-space hydrocephalus, *iNPH *idiopathic normal-pressure hydrocephalus, *MCI *mild cognitive impairment, *TUG *Timed Up and Go

Overall, 221 individuals were excluded, as detailed in Fig. 2 and Additional file 1: Tables S1–S4. Thus, 1,356 participants (mean age: 73.8 ± 6.2 years; 834 women [61.5%]) were included in the analysis. Table 1 shows the demographic and clinical characteristics of the study population.

**Table 1:** Demographic and clinical characteristics of participants

	All individuals (*n* = 1,356)	DESH (*n* = 25)	non-DESH (*n* = 1,331)	DESH vs. non-DESH
				*P*-value
Age, years	73.8 (6.2)	79.4 (7.5)	73.7 (6.1)	< 0.0001*
Female sex	834 (61.5%)	13 (52.0%)	821 (61.7%)	0.3242
Education (≤ 9 years)	354 (26.1%)	5 (20.0%)	349 (26.2%)	0.4829
Hypertension	972 (71.7%)	21 (84.0%)	951 (71.5%)	0.1676
Diabetes mellitus	209 (15.4%)	5 (20.0%)	204 (15.3%)	0.5214
Dyslipidemia	692 (51.0%)	9 (36.0%)	683 (51.3%)	0.1291
Atrial fibrillation	81 (6.0%)	2 (8.0%)	79 (5.9%)	0.6661
Coronary artery disease	70 (5.2%)	4 (16.0%)	66 (5.0%)	0.0134*
Heart failure	19 (1.4%)	0 (0.0%)	19 (1.4%)	0.5474
BMI	23.5 (3.3)	23.2 (2.7)	23.5 (3.3)	0.6760
History of smoking	354 (26.1%)	5 (20.0%)	349 (26.2%)	0.4829
MMSE	27.4 (2.5)	25.9 (2.9)	27.4 (2.4)	0.0021*
TUG	8.6 (1.7)	10.1 (2.0)	8.6 (1.7)	< 0.0001*
Cognitive impairment^a^	221 (16.3%)	8 (32.0%)	213 (16.0%)	0.0319*
Gait disturbance^b^	58 (4.3%)	4 (16.0%)	54 (4.1%)	0.0034*
Imaging				
MRI scanner (Philips)	901 (66.4%)	17 (68.0%)	884 (66.4%)	0.8680
Fazekas score^c^	2.1 (2.1)	3.7 (1.8)	2.1 (2.1)	0.0002*
Lacunar infarction	300 (22.1%)	13 (52.0%)	287 (21.6%)	0.0003*
Microbleeds	187 (13.8%)	6 (24.0%)	181 (13.6%)	0.1351
Perivascular space	0.0001 (0.00005)	0.0001 (0.00005)	0.0001 (0.00005)	0.5991
VS	0.0335 (0.0088)	0.0527 (0.0092)	0.0331 (0.0084)	< 0.0001*
SF	0.0136 (0.0022)	0.0180 (0.0029)	0.0136 (0.0021)	< 0.0001*
SHM	0.0371 (0.0074)	0.0206 (0.0067)	0.0374 (0.0071)	< 0.0001*

Data are *n* (%) or mean (SD). The volume of each DESH-related region was normalized to the total intracranial volume

^*^Significance at level *P* < 0.05

^a^MCI

^b^TUG > 12.0 s

^c^The sum of periventricular and deep WMH scores, ranging from 0 to 6

*DESH* disproportionately enlarged subarachnoid-space hydrocephalus, *SF* Sylvian fissure, *SHM* subarachnoid space at high convexity and midline, *VS* ventricular system

In the visual DESH assessment, 214 (15.8%) participants had at least one DESH feature. Further, 118 (8.7%), 86 (6.3%), and 131 (9.6%) participants had ventriculomegaly, enlarged SF, and tight SHM, respectively. Moreover, 25 (1.8%) participants had DESH; of these, 10 (0.7%) and 15 (1.1%) participants were classified as having possible iNPH with MRI support and AVIM, respectively (Fig. 2).

Correlations of volumes of DESH-related regions with their visual assessments are provided in Additional file 1: Figure S1 (see Additional file 1). VS volume exhibited a positive linear correlation with EI (*r* = 0.695, *P* < 0.0001). SF volume was positively correlated with its visual rating scale (*rs* = 0.412, *P* < 0.0001), and SHM volume was negatively correlated with its visual rating scale (*rs* = -0.434, *P* < 0.0001).

The DESH and non-DESH groups are compared in Table 1. Compared to the non-DESH group, the DESH group was older and had a higher prevalence of coronary artery disease, lower MMSE score, higher TUG and Fazekas scores, and more lacunar infarctions. VS and SF were higher, whereas the SHM was lower in the DESH group than in the non-DESH group.

Individuals without DESH (*n* = 1331) were compared in two groups, the MCI group (*n* = 213) and the normal cognitive (NC) group (*n* = 1118) (see Additional file 1: Table S6). Compared to the NC group, the MCI group was older and had more men, lower education, a higher prevalence of atrial fibrillation, lower MMSE score, higher TUG and Fazekas scores, and more lacunar infarctions. Furthermore, VS and SF volumes were higher, whereas SHM volume was lower in the MCI group than in the NC group.

**Fig. 3:**
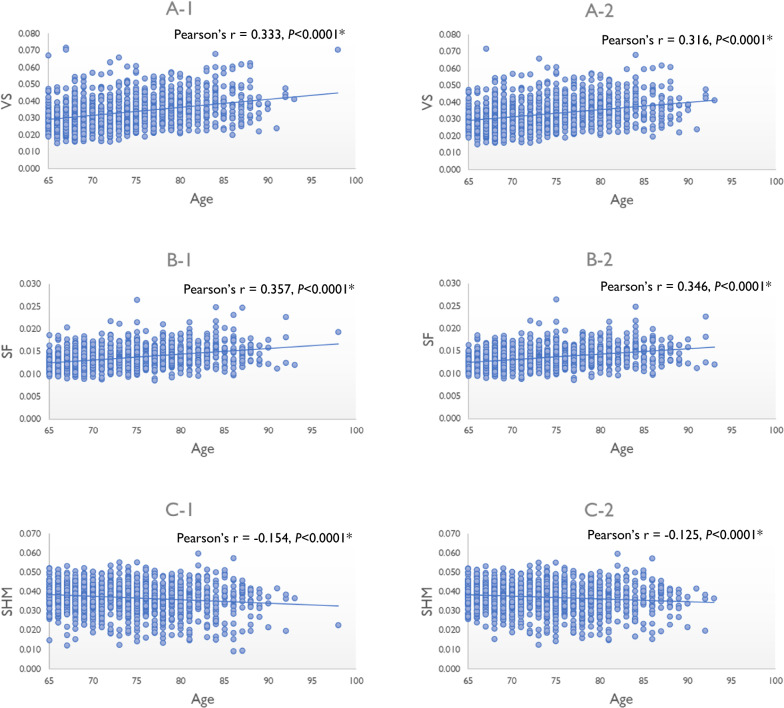
Correlation of DESH-related regions with age. Scatter plots showing the relationships between the volume of each DESH-related region and age. **A-1** VS and age in all individuals. **A-2** VS and age in individuals without DESH. (**B-1)** SF and age in all individuals. **B-2** SF and age in individuals without DESH. **C-1** SHM and age in all individuals. **C-2** SHM and age in individuals without DESH. The volume of each DESH-related region was normalized to the total intracranial volume. *The significance level was set at *P* < 0.05. *BMI *body mass index, *DESH *disproportionately enlarged subarachnoid-space hydrocephalus, *SF *Sylvian fissure, *SHM *subarachnoid space at high convexity and midline, *VS *ventricular system

The correlation analysis results are illustrated in scatterplot diagrams to represent the relationships between the volume of each DESH-related region and age (Fig. 3). Correlation analysis indicated that the VS and SF volumes were positively correlated with age (Fig. 3A-1 and B-1). In contrast, the SHM volume was negatively correlated with age (Fig. 3C-1) in all individuals. These correlations remained significant after controlling for confounding factors (Table 2). Furthermore, these associations remained significant for all DESH-related regions, even when individuals with DESH were excluded (Fig. 3A-2, B-2, C-2, and Table 2). The associations between the volume of brain structures and age are provided (see Additional file 1: Table S7). After adjusting for confounding factors, the volumes of all brain structures, except for the caudal anterior cingulate, frontal pole, temporal pole, pericalcarine, caudate, and pallidum, were negatively correlated with age.

**Table 2:** Multivariate analysis of DESH-related regions

VS				
Variables	All individuals (*n* = 1,356)		Individuals without DESH (*n* = 1,331)	
	β_STD_	*P*-value	β_STD_	*P*-value
Age	0.3199	< 0.0001*	0.3082	< 0.0001*
Female sex	−0.2343	< 0.0001*	−0.2423	< 0.0001*
Education (≤ 9 years)	−0.0243	0.3478	−0.0126	0.6329
Hypertension	−0.0080	0.7602	−0.0085	0.7486
Diabetes mellitus	0.0447	0.0772	0.0391	0.1279
Dyslipidemia	−0.0428	0.0913	−0.0429	0.0953
Atrial fibrillation	0.0146	0.5592	0.0186	0.4644
Coronary artery disease	0.0225	0.3718	0.0072	0.7777
Heart failure	0.0094	0.7067	0.0160	0.5306
BMI	−0.0151	0.5538	−0.0126	0.6282
History of smoking	−0.0129	0.6835	−0.0134	0.6785
Fazekas score	0.1473	< 0.0001*	0.1504	< 0.0001*
Lacunar infarction	−0.0571	0.0692	−0.0803	0.0117*
Microbleeds	0.0112	0.6673	0.0078	0.7668
Perivascular space	0.0425	0.0888	0.0411	0.1047

SF				
Variables	All individuals (*n* = 1,356)		Individuals without DESH (*n* = 1,331)	
	β_STD_	*P*-value	β_STD_	*P*-value
Age	0.3280	< 0.0001*	0.3152	< 0.0001*
Female sex	−0.0704	0.0310*	−0.0588	0.0756
Education (≤ 9 years)	0.0104	0.6955	0.0225	0.4029
Hypertension	0.0206	0.4416	0.0123	0.6488
Diabetes mellitus	0.0228	0.3766	0.0231	0.3771
Dyslipidemia	−0.0299	0.2468	-0.0213	0.4157
Atrial fibrillation	0.0539	0.0355*	0.0767	0.0031*
Coronary artery disease	0.0189	0.4622	0.0041	0.8737
Heart failure	0.0166	0.5166	0.0212	0.4129
BMI	−0.0036	0.8888	0.0046	0.8623
History of smoking	0.0446	0.1677	0.0490	0.1361
Fazekas score	0.0707	0.0259*	0.0629	0.0503
Lacunar infarction	0.0046	0.8849	0.0072	0.8251
Microbleeds	0.0093	0.7268	0.0169	0.5295
Perivascular space	0.0456	0.0733	0.0538	0.0372*

SHM				
Variables	All individuals (*n* = 1,356)		Individuals without DESH (*n* = 1,331)	
	β_STD_	*P*-value	β_STD_	*P*-value
Age	−0.1104	0.0002*	−0.0833	0.0063*
Female sex	0.0717	0.0363*	0.0650	0.0619
Education (≤ 9 years)	0.0015	0.9563	−0.0158	0.5765
Hypertension	−0.0632	0.0246*	−0.0624	0.0288*
Diabetes mellitus	−0.0006	0.9809	0.0061	0.8234
Dyslipidemia	−0.0052	0.8469	−0.0159	0.5635
Atrial fibrillation	0.0064	0.8105	−0.0078	0.7741
Coronary artery disease	−0.0124	0.6459	0.0041	0.8808
Heart failure	−0.0120	0.6538	−0.0187	0.4930
BMI	−0.0311	0.2559	−0.0360	0.1963
History of smoking	−0.0306	0.3680	−0.0355	0.3045
Fazekas score	−0.1601	< 0.0001*	−0.1525	< 0.0001*
Lacunar infarction	0.0242	0.4735	0.0352	0.3024
Microbleeds	0.0074	0.7920	0.0063	0.8242
Perivascular space	−0.0822	0.0022*	−0.0888	0.0011*

*The significance level was *P* < 0.05

*BMI* body mass index, *DESH* disproportionately enlarged subarachnoid-space hydrocephalus, *SF* Sylvian fissure, *SHM* subarachnoid space at high convexity and midline, *VS* ventricular system

**Fig. 4:**
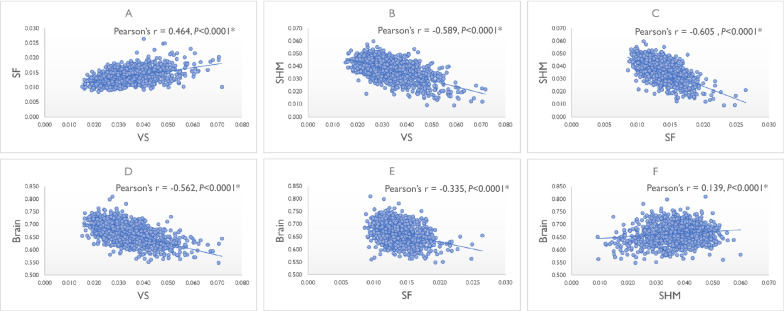
Correlation between the VS, SF, and SHM volumes and brain volume. **A** VS and SF. **B** VS and SHM. **C** SF and SHM. **D**VS and brain. **E** SF and brain. **F** SHM and brain. *The significance level was set at *P* < 0.05. *SF *Sylvian fissure, *SHM *subarachnoid space at high convexity and midline, *VS *ventricular system

Scatterplot diagrams showing the association between the VS, SF, and SHM volumes and total brain volume are provided in Fig. 4. VS exhibited a positive linear correlation with SF (Fig. 4A) and a negative linear correlation with SHM (Fig. 4B). SF was negatively correlated with SHM (Fig. 4C). Total brain volume was negatively correlated with VS volume (Fig. 4D) and SF volume (Fig. 4E) and positively correlated with SHM volume (Fig. 4F). These correlations remained significant, even when individuals with DESH were excluded (see Additional file 1: Figure S2). To examine the association between the SHM volume and brain atrophy in detail, we investigated the association between the volume of SHM and its adjacent parietal cortices (the sum of inferior parietal, isthmus cingulate, postcentral, posterior cingulate, precuneus, superior parietal, and supramarginal). SHM volume was positively correlated with the volume of parietal cortices (see Additional file 1: Figure S3); SHM did not expand even if parietal cortices atrophied. This association persisted even when individuals with DESH were excluded (see Additional file 1: Figure S3).

In the multivariate analysis of DESH-related regions, certain variables, in addition to age, were significantly associated with DESH-related regions (Table 2). Male sex, higher Fazekas score, and lower prevalence of lacunar infarction were associated with an enlarged VS (only lacunar infarction did not reach significance in the overall group). Male sex, higher prevalence of atrial fibrillation, higher Fazekas scores, and larger perivascular space were associated with an enlarged SF (sex reached significance only in the overall group, Fazekas score did not reach significance in individuals without DESH, and perivascular space reached significance only in individuals without DESH). Male sex, a higher prevalence of hypertension, higher Fazekas score, and larger perivascular space were associated with lower SHM in all groups (sex reached significance only in the overall group).

The results of the hierarchical multiple regression analysis for the MMSE and TUG scores with brain structures are presented in Tables 3 and 4, respectively. For each analysis, the top twenty brain structures are shown in descending order of ∆*R*^2^. All data are shown in Additional file 1: Tables S8, S9. When analyzed in all individuals, SHM and VS were associated with the MMSE scores and had β_STD_ coefficients of 0.0993 and −0.0916, respectively. In individuals without DESH, SHM and VS were associated with the MMSE scores and had β_STD_ coefficients of 0.0910 and −0.0932, respectively (Table 3). In all individuals, no DESH-related regions were associated with the TUG scores, whereas 16 brain regions were significantly correlated with the TUG scores. In individuals without DESH, no DESH-related regions correlated with the TUG scores, whereas 11 brain regions were significantly correlated with the TUG scores (Table 4). All data met the assumptions required for regression analysis.

**Table 3:** Results of hierarchical multiple regression analysis for MMSE with brain structures

	All individuals (*n* = 1,356)					Individuals without DESH (*n* = 1,331)			
	Brain structure	β_STD_	*P*-value	ΔR^2^		Brain structure	β_STD_	*P*-value	ΔR^2^
1	SHM	0.0993	0.0002*	0.0090	1	SHM	0.0910	0.0006*	0.0076
2	VS	−0.0916	0.0010*	0.0068	2	VS	−0.0932	0.0009*	0.0071
3	Accumbens	0.0919	0.0016	0.0062	3	Accumbens	0.0914	0.0019	0.0062
4	Hippocampus	0.0904	0.0029	0.0056	4	Middle temporal	0.0901	0.0020	0.0061
5	Middle temporal	0.0852	0.0033	0.0054	5	Precuneus	0.0926	0.0024	0.0059
6	SF	−0.0783	0.0042	0.0052	6	Hippocampus	0.0926	0.0024	0.0059
7	Precuneus	0.0863	0.0043	0.0051	7	Lateral occipital	0.0807	0.0051	0.0050
8	Lateral occipital	0.0808	0.0047	0.0050	8	Amygdala	0.0819	0.0051	0.0050
9	Supramarginal	0.0799	0.0052	0.0049	9	Banks sts	0.0822	0.0053	0.0050
10	Amygdala	0.0811	0.0052	0.0049	10	Supramarginal	0.0797	0.0056	0.0049
11	Lingual	0.0708	0.0107	0.0041	11	Lingual	0.0725	0.0096	0.0043
12	Transverse temporal	0.0685	0.0125	0.0039	12	SF	-0.0704	0.0104	0.0042
13	Superior temporal	0.0704	0.0161	0.0036	13	Superior temporal	0.0724	0.0141	0.0039
14	Lateral orbitofrontal	0.0658	0.0196	0.0034	14	Superior frontal	0.0756	0.0143	0.0039
15	Superior frontal	0.0712	0.0200	0.0034	15	Inferior parietal	0.0714	0.0188	0.0035
16	Inferior parietal	0.0675	0.0254	0.0031	16	Inferior temporal	0.0649	0.0220	0.0034
17	Banks sts	0.0647	0.0263	0.0031	17	Transverse temporal	0.0632	0.0225	0.0033
18	Inferior temporal	0.0595	0.0347	0.0028	18	Lateral orbitofrontal	0.0641	0.0236	0.0033
19	Precentral	0.0711	0.0367	0.0028	19	Precentral	0.0630	0.0669	0.0022
20	Entorhinal	0.0487	0.0659	0.0021	20	Entorhinal	0.0469	0.0784	0.0020

After controlling for age, sex, education, hypertension, diabetes mellitus, dyslipidemia, atrial fibrillation, coronary artery disease, heart failure, BMI, history of smoking, MRI scanner, Fazekas score, lacunar infarction, microbleeds and perivascular space (Block 1), each brain structure was entered in Block 2. For each analysis, the top 20 brain structures are shown in descending order of ∆R^2^

^*^The significance level was *P* < 0.0011 to correct for 46 modeling analyses

*BMI* body mass index, *DESH* disproportionately enlarged subarachnoid-space hydrocephalus, *MMSE* Mini-Mental State Examination, *SF* Sylvian fissure, *SHM* subarachnoid space at high convexity and midline, *VS* ventricular system

**Table 4:** Results of hierarchical multiple regression analysis for TUG with brain structures

	All individuals (*n* = 1,356)					Individuals without DESH (*n* = 1,331)			
	Brain structure	β_STD_	*P*-value	ΔR^2^		Brain structure	β_STD_	*P*-value	ΔR^2^
1	Precentral	−0.1534	< 0.0001*	0.0128	1	Precentral	−0.1433	< 0.0001*	0.0112
2	Pars orbitalis	−0.1188	< 0.0001*	0.0120	2	Pars orbitalis	−0.1091	< 0.0001*	0.0101
3	Lateral orbitofrontal	−0.1227	< 0.0001*	0.0119	3	Lateral orbitofrontal	−0.1115	< 0.0001*	0.0100
4	Lingual	−0.1127	< 0.0001*	0.0104	4	Lingual	−0.1078	0.0001*	0.0095
5	Accumbens	−0.1163	< 0.0001*	0.0100	5	Superior parietal	−0.1148	0.0002*	0.0088
6	Superior parietal	−0.1216	< 0.0001*	0.0099	6	Accumbens	−0.1073	0.0003*	0.0085
7	Insula	−0.1123	0.0001*	0.0093	7	Insula	−0.1013	0.0006*	0.0076
8	Superior frontal	−0.1134	0.0002*	0.0086	8	Precuneus	−0.1040	0.0007*	0.0074
9	Superior temporal	−0.1082	0.0002*	0.0086	9	Superior frontal	−0.1050	0.0007*	0.0074
10	Supramarginal	−0.1049	0.0002*	0.0085	10	Middle temporal	−0.0983	0.0008*	0.0073
11	Posterior cingulate	−0.1032	0.0003*	0.0084	11	Superior temporal	−0.0970	0.0010*	0.0070
12	Middle temporal	−0.1056	0.0003*	0.0083	12	Supramarginal	−0.0934	0.0012	0.0068
13	Precuneus	−0.1075	0.0004*	0.0080	13	Inferior temporal	−0.0918	0.0012	0.0067
14	Pallidum	−0.0958	0.0005*	0.0076	14	Pallidum	−0.0878	0.0018	0.0063
15	Hippocampus	−0.1005	0.0009*	0.0069	15	Hippocampus	−0.0955	0.0018	0.0063
16	Inferior temporal	−0.0923	0.0010*	0.0068	16	Posterior cingulate	−0.0851	0.0028	0.0058
17	Transverse temporal	−0.0892	0.0011	0.0067	17	Corpus callosum	−0.0857	0.0039	0.0054
18	Corpus callosum	−0.0900	0.0022	0.0059	18	Banks sts	−0.0850	0.0040	0.0053
19	Banks sts	−0.0882	0.0024	0.0058	19	Caudate	−0.0822	0.0056	0.0050
20	Rostral anterior cingulate	−0.0790	0.0028	0.0056	20	Transverse temporal	−0.0736	0.0080	0.0045

After controlling for age, sex, education, hypertension, diabetes mellitus, dyslipidemia, atrial fibrillation, coronary artery disease, heart failure, BMI, history of smoking, MRI scanner, Fazekas score, lacunar infarction, microbleeds and perivascular space (Block 1), each brain structure was entered in Block 2. For each analysis, the top 20 brain structures are shown in descending order of ∆R^2^

^*^The significance level was *P* < 0.0011 to correct for 46 modeling analyses

*BMI* body mass index, *DESH* disproportionately enlarged subarachnoid-space hydrocephalus, *MMSE,* Mini-Mental State Examination, *SF* Sylvian fissure, *SHM* subarachnoid space at high convexity and midline, *TUG* Timed Up and Go, *VS* ventricular system

## Discussion

This research focused on age-related CSF dynamics, measuring the volume of CSF space in a large sample of community-dwelling older people. Regarding the relationships between the volume of each CSF space and age, VS and SF volumes increased with age, as expected. However, the SHM volume did not increase and tended to decrease with age. These three CSF spaces correlated with each other; VS and SF volumes were positively correlated, and both were negatively correlated with SHM volume. These correlations remained even after excluding individuals with DESH. The pattern of age-related changes in the three CSF spaces shown in the community-dwelling older people were similar to that of DESH findings in patients with iNPH (tight high-convexity and medial subarachnoid spaces, enlarged SF, and ventriculomegaly). These results suggested that the brains of older adults gradually undergo DESH-like changes with aging; that is, DESH-like morphological changes are not rare phenomena but can occur universally in the aged human brain due to altered CSF dynamics.

CSF space is understood to expand as brain atrophy progresses. We found that the volumes of VS and SF increased as the whole brain volume decreased. However, it is striking that the SHM volume was positively correlated with the whole brain volume (although the absolute value of the correlation coefficient was small); that is, SHM did not expand even if brain atrophy progressed. SHM volume was also positively correlated with the volumes of adjacent parietal cortices. These results remained unchanged, even after excluding individuals with DESH. The seemingly paradoxical relationship between brain atrophy and SHM volume suggests that brain atrophy may have some effect on CSF dynamics, although the detailed mechanism is unknown. Further research is needed on the relationship between brain atrophy and CSF dynamics. The present finding, which overturns the conventional belief that SHM expands as the brain atrophies, can provide significant implications for practice in dementia. The expansion of SHM on MRI does not necessarily indicate parietal lobe atrophy.

Previous studies have shown that DESH exists among community-dwelling older people and that its prevalence increases with age [10, 13]. However, to the best of our knowledge, the present study is the first to show that the human brain generally changes toward a “DESH-like” morphology with aging, even in older people without dementia. Why has this significant finding not been ever noticed? The discovery of DESH originated from iNPH research [11, 12]; thus, DESH-like morphological changes have not been well studied in the context of normal brain aging. In fact, no study has directly quantified the volume of CSF space in large-scale samples or investigated the association between CSF volume and age. Additionally, it seems that age-related SHM volume reduction has often been overlooked because the relatively minor SHM narrowing seen in healthy older people can be regarded as not atrophic or as brain morphological changes within normal limits on visual assessment. Moreover, post-mortem studies are unsuitable for research on age-related CSF volume changes. In this study, the measurement of both CSF space and brain volume using MRI in a large sample of community-dwelling older people made it possible to identify the trend toward a DESH-like morphology with aging.

To investigate the clinical significance of this age-related morphological change, we examined the relationship between age-related DESH-like morphological changes and the clinical symptoms of iNPH (e.g., cognitive and gait functions). MMSE scores were associated with SHM and VS, which suggested that decreased SHM volume and increased VS volume have a prominent role in cognitive decline. This association did not change in individuals without DESH, suggesting that decreased SHM volume and increased VS volume are associated with cognitive decline, even without meeting the definition of DESH. Few studies have shown an association between quantified DESH imaging patterns and cognitive dysfunction in the absence of iNPH [15, 16]. In our study, similar findings were replicated using a different method directly quantifying the volumes of the DESH-related regions. In the present study, in individuals without DESH, the MCI group had a lower SHM volume, larger VS volume, and lower MMSE scores than those in the cognitively unimpaired group. These findings suggest that some people in the MCI group have cognitive decline due to reduced SHM volume and increased VS volume, independent of brain volume reduction. For all individuals and individuals without DESH, the top ten brain regions—listed in descending order of ∆*R*^2^ associated with the MMSE score (i.e., hippocampus, middle temporal, precuneus, lateral occipital, supramarginal, and amygdala)—have been reported as atrophic regions in MCI, Alzheimer’s disease, and dementia with Lewy bodies [30–34]. The present results suggest that DESH-like morphological changes as well as brain atrophy are important conditions associated with cognitive decline in older adults.

In this study, the DESH group had a significantly higher TUG scores than the non-DESH group. However, no DESH-related regions were associated with the TUG score. These results suggest that gait disturbance appears only when the DESH brain morphology is completed. A study reported no significant differences in gait velocity between the HCTS group and the non-HCTS group in the general population [15], which agrees with the results of this study. Thus, unlike cognitive dysfunction, gait disturbance does not appear only with decreasing SHM volume; it may also require increased VS and SF volumes. The results of this study suggest that gait disturbance appears later than cognitive dysfunction. This difference in onset may explain the difference in the treatment responsiveness of iNPH in that improvement is more likely for gait disturbance than for cognitive dysfunction.

In the present study, 1.8% of individuals had DESH and 1.1% had AVIM. Past studies have reported a DESH prevalence of 1.0% [13] and an AVIM prevalence of 1.0% [35]. Although the evaluation methods for DESH and AVIM differed between the aforementioned studies and our study, the prevalence of each in community-dwelling older populations was similar. The DESH prevalence was low, although the human brain generally changes toward a DESH-like morphology with aging, which suggests that DESH might be an accelerated aging stage. Awareness of the DESH-like morphological changes with age is important owing to the early detection and treatment of iNPH as well as early interventions preventing progression toward DESH. Focusing on CSF dynamics with aging and developing methods to maintain normal CSF dynamics may contribute to preservation of cognitive and gait functions in older people.

It is unclear why DESH-like brain morphological changes occur with age; however, based on previous findings [5, 36, 37], age-related changes in CSF dynamics in the human brain may occur via various mechanisms. Aged mice reportedly have a disruption in meningeal lymphatic function [5], and advancing age was associated with a decline in the efficiency of CSF-interstitial fluid exchange in rodents [36]. Ventriculomegaly in the human brain occurs partly because of age-related timing dissociation between the superficial and deep venous system [37]. The factors associated with the DESH-related regions in our study, such as hypertension, perivascular space and WMH, may be related to their pathophysiology. Hypertension decreases the net flow of CSF in the perivascular spaces of rodents, suggesting a decline in glymphatic function [38]. In our study, a higher prevalence of hypertension and a larger perivascular space were associated with lower SHM; therefore, hypertension may reduce the SHM volume via impaired glymphatic function. SHM volume reduction was more strongly associated with WMH than with age, and the pathophysiology of SHM may differ from that of VS and SF, which were most strongly associated with age. The background pathophysiology of WMH is heterogeneous [39]; therefore, future investigations of the pathophysiology of WMH that contributes to SHM volume reduction are warranted.

The main strength of this study is that it directly quantified the CSF volume of all three DESH-related regions in a large sample of community-dwelling older individuals by using independently developed VBM software. The reliability of the data was high because the software has been used to study the pathology of iNPH and has been confirmed to accurately assess DESH [25, 26].

This study has some limitations. First, it was a cross-sectional analysis; thus, the findings do not indicate a causal relationship between DESH-related regions and associated factors. Future longitudinal studies will be required for this purpose. Second, the participants in this study were older people (aged  ≥ 65 years); therefore, it remains unclear when DESH-like morphological changes, especially SHM volume reduction, begin. Further research is needed to clarify this point. Third, we prepared VOI templates for high parietal convexity/midline when using VBM software; therefore, how the subarachnoid space adjacent to other brain regions, such as the frontal lobe, changes with age is unclear. Finally, MRI was conducted using two different devices, and we included the scanner type in the multivariable regression analysis as a covariate to exclude the bias in the results.

## Conclusions

The volume of high-convexity and medial subarachnoid spaces tends to decrease with age, and the human brain continuously progresses toward a DESH-like morphology with aging in community-dwelling older persons. In addition, this morphological change is an important condition associated with cognitive decline in older adults. DESH might be an accelerated aging stage; thus, more attention should be focused on CSF dynamics in the aging process. Developing methods to maintain normal CSF dynamics may improve brain health in older people.

## Supplementary Information

**Additional file 1: Figure S1. **Correlation of volumes of DESH-related regions with their visual assessments. **Figure S2.** Correlation among the volume of VS, SF, SHM and brain in individuals without DESH. **Figure S3. **Correlation between the volume of SHM and parietal cortices. **Table S1.** Details of dementia subtype. **Table S2. **The characteristics of the participants with severe gait disturbance. **Table S3.** The list of participants with unsuitable MRI for quantitative analysis. **Table S4.** Proportion of missing data for variables recorded in this study. **Table S5.** The list of brain regions whose volumes were calculated by FreeSurfer. **Table S6.** Comparison between characteristics of MCI group and cognitively normal group in individuals without DESH. **Table S7.** Association of volume of brain structures with age. **Table S8.** Results of hierarchical multiple regression analysis for MMSE with brain structures. **Table S9.** Results of hierarchical multiple regression analysis for TUG with brain structures.

## Data Availability

The raw data are not openly available to protect the confidentiality of the participants and to comply with the terms of participant consent. Requests related to the raw data should be addressed to the corresponding author.

## Abbreviations

*AVIM*: Asymptomatic ventriculomegaly with features of idiopathic normal-pressure hydrocephalus on MRI
*BMI*: Body mass index
*CSF*: Cerebrospinal fluid
*DESH*: Disproportionately enlarged subarachnoid-space hydrocephalus
*EI*: Evans index
*FLAIR*: Fluid-attenuated inversion recovery
*GM*: Gray matter
*HCTS*: High-convexity tight sulci
*iNPH*: Idiopathic normal-pressure hydrocephalus
*MCI*: Mild cognitive impairment
*MRI*: Magnetic resonance imaging
*MMSE*: Mini-Mental State Examination
*NC*: Normal cognitive
*SF*: Sylvian fissure
*SHM*: Subarachnoid space at high convexity and midline
*SWI*: Susceptible weighting image
*TUG*: Timed up and go
*VOI*: Voxels of interest
*VBM*: Voxel-based morphometry
*VS*: Ventricular system
*WM*: White matter
*WMH*: White matter hyperintensity
