# Supplementary material for: Impact of age on the cerebrospinal fluid spaces: high-convexity and medial subarachnoid spaces decrease with age
Source: Fluids Barriers CNS. 2022 Oct 28;19:82. doi: 10.1186/s12987-022-00381-5 (PMC9615391; doi:10.1186/s12987-022-00381-5)
Supplement: Supplementary file 1 — Additional file 1: Figure S1. Correlation of volumes of DESH-related regions with their visual assessments. Figure S2. Correlation among the volume of VS, SF, SHM and brain in individuals without DESH. Figure S3. Correlation between the volume of SHM and parietal cortices. Table S1. Details of dementia subtype. Table S2. The characteristics of the participants with severe gait disturbance. Table S3. The list of participants with unsuitable MRI for quantitative analysis. Table S4. Proportion of missing data for variables recorded in this study. Table S5. The list of brain regions whose volumes were calculated by FreeSurfer. Table S6. Comparison between characteristics of MCI group and cognitively normal group in individuals without DESH. Table S7. Association of volume of brain structures with age. Table S8. Results of hierarchical multiple regression analysis for MMSE with brain structures. Table S9. Results of hierarchical multiple regression analysis for TUG with brain structures. [file 12987_2022_381_MOESM1_ESM.pdf]

Supplementary material for
Impact of age on the cerebrospinal fluid spaces: high-convexity and medial subarachnoid spaces decrease with age

Table of contents

Figure S1: Correlation of volumes of DESH-related regions with their visual assessments 2
Figure S2: Correlation among the volume of VS, SF, SHM and brain in individuals without DESH 3
Figure S3: Correlation between the volume of SHM and parietal cortices 4
Table S1: Details of dementia subtype 5
Table S2: The characteristics of the participants with severe gait disturbance 6
Table S3: The list of participants with unsuitable MRI for quantitative analysis 7
Table S4: Proportion of missing data for variables recorded in this study 9
Table S5: The list of brain regions whose volumes were calculated by FreeSurfer 10
Table S6: Comparison between characteristics of MCI group and cognitively normal group in individuals without DESH 13
Table S7: Association of volume of brain structures with age 15
Table S8: Results of hierarchical multiple regression analysis for MMSE with brain structures 17
Table S9: Results of hierarchical multiple regression analysis for TUG with brain structures 18

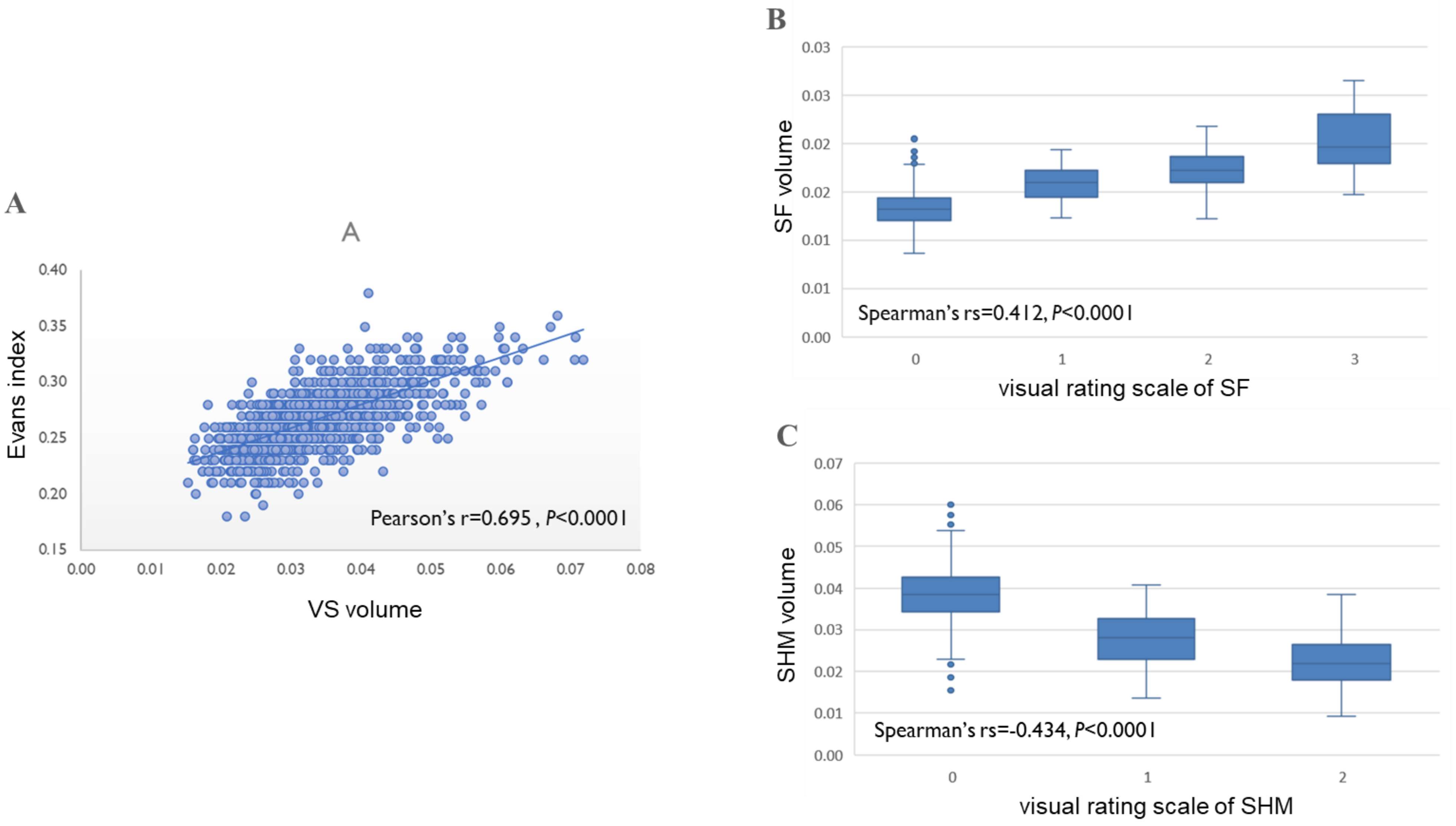

Figure S1: Correlation of volumes of DESH-related regions with their visual assessments

A: Scatter plot showing the relationship between Evans index and the VS volume. B: Box-plot diagram showing the relationship between the visual rating scales of SF and the SF volume. C: Box-plot diagram showing the relationship between the visual rating scales of SHM and the SHM volume. DESH=Disproportionately enlarged subarachnoid-space hydrocephalus; SF=Sylvian fissure; SHM=Sulci at high convexity and the midline; VS=Ventricle system

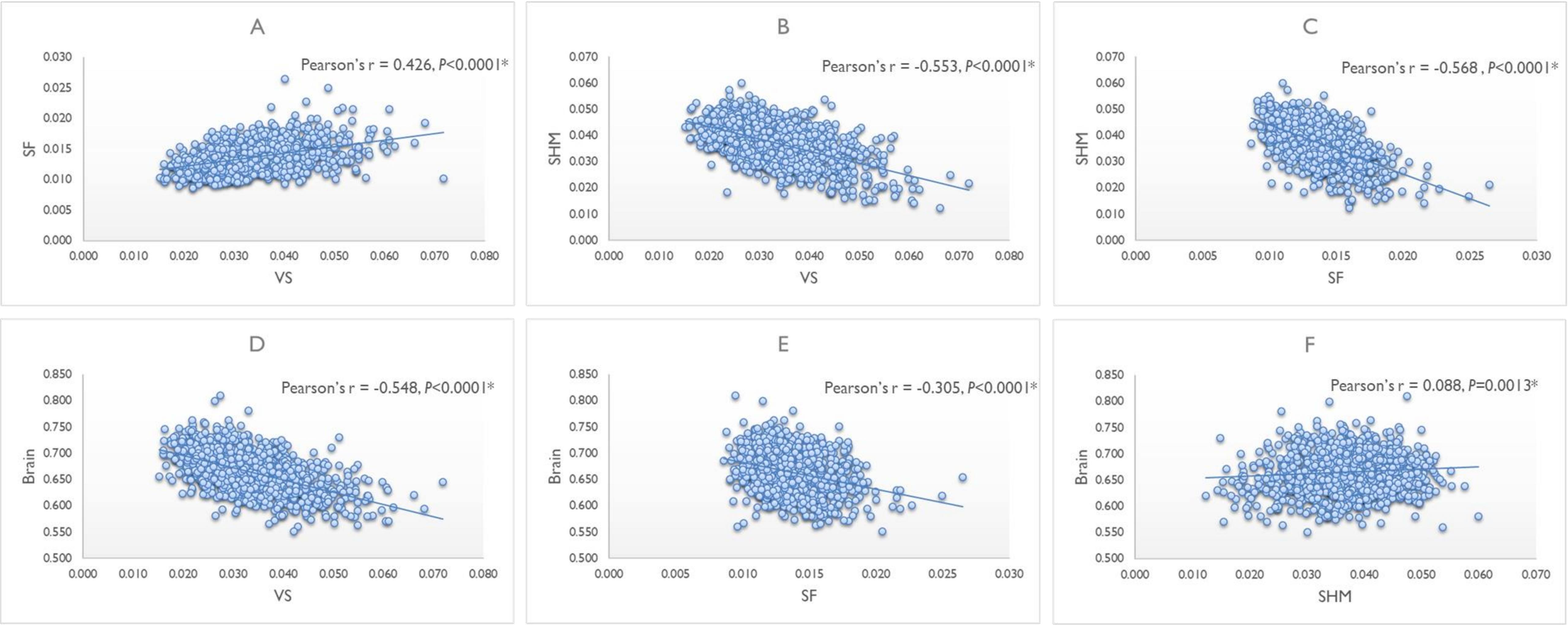

Figure S2: Correlation among the volume of VS, SF, SHM and brain in individuals without DESH
A: VS and SF, B: VS and SHM, C: SF and SHM, D: VS and brain, E: SF and brain, F: SHM and brain
*The significance level was *P*<0.05

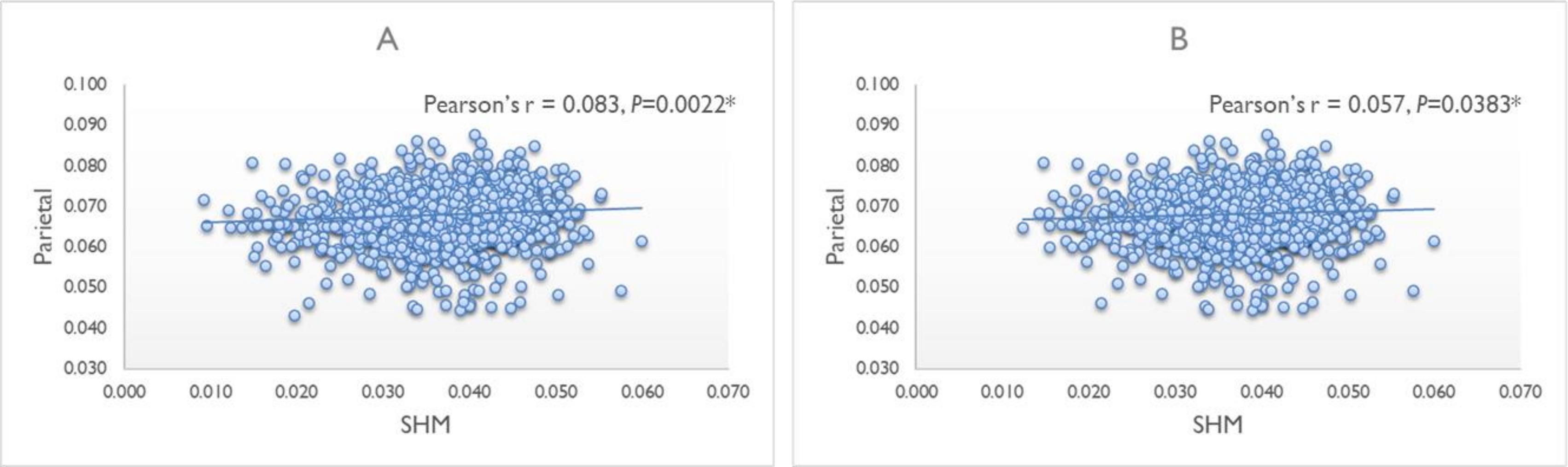

Figure S3: Correlation between the volume of SHM and parietal cortices
A: SHM and parietal cortices in all participants (n=1356), B: SHM and parietal cortices in participants without DESH (n=1331)
*The significance level was *P*<0.05

Table S1: Details of dementia subtype

Dementia subtype	Number of participants	Frequency (%)
AD (isolated)	47	81.0
VaD (isolated)	5	8.6
DLB (isolated)	3	5.2
iNPH (isolated)	1	1.7
Mixed type	2	3.4
AD+iNPH	1	-
VaD+CO toxication	1	-

AD=Alzheimer's disease; VaD=Vascular dementia; DLB=Dementia with Lewy bodies; iNPH=Idiopathic normal pressure hydrocephalus; CO=Carbon monoxide

Table S2: The characteristics of the participants with severe gait disturbance

age	sex	TUG	MMSE	Education (≤ 9 years)	HT	DM	Dyslipidaemia	AF	CAD	HF	BMI	Smoking	Fazekas score [a)]	Lacunar infarction	Microbleeds	Perivascular space	MRI scanner
70	Male	26.2	28	+	+	-	-	-	-	-	26.9	-	3	+	-	0.000036	Philips
71	Female	21.7	20	+	+	-	-	+	-	-	26.0	-	2	-	-	0.000096	GE
72	Female	31.9	29	-	+	-	-	-	-	-	17.7	-	4	+	-	0.000201	Philips
78	Male	18.4	13	+	+	-	+	-	-	-	24.0	+	6	+	+	0.000085	Philips
80	Female	18.3	29	-	+	-	+	-	-	-	21.7	-	0	-	-	0.000050	GE
81	Male	24.0	21	+	+	+	-	-	-	-	19.1	-	0	-	-	0.000131	Philips
83	Female	18.8	25	+	+	-	+	-	+	-	27.1	-	4	-	-	0.000108	Philips
85	Male	28.5	24	+	na	na	na	-	-	-	24.2	+	6	+	+	0.000072	Philips
85	Female	17.3	28	-	+	-	-	-	+	-	19.9	-	4	-	-	0.000079	Philips
85	Female	26.5	27	-	+	-	-	-	-	-	37.8	-	5	+	-	0.000146	GE
87	Female	17.9	28	+	-	-	-	+	-	-	19.4	-	4	+	-	0.000097	Philips
87	Female	19.0	27	+	+	-	+	-	-	-	26.7	-	5	+	+	0.000158	Philips
88	Female	27.0	27	+	+	+	+	+	-	-	27.8	-	6	+	-	0.000292	Philips
90	Female	23.7	29	+	-	+	+	+	-	-	26.7	-	0	-	-	0.000100	Philips
91	Female	40.6	23	-	+	+	+	-	-	-	24.5	-	6	-	+	0.000101	GE
92	Female	29.6	22	+	+	-	+	-	-	-	26.1	-	3	+	-	0.000113	GE
92	Male	24.7	15	+	+	-	-	-	-	-	22.4	+	6	+	-	0.000152	Philips
92	Male	17.3	27	-	+	-	-	-	-	-	18.1	+	4	+	+	0.000346	Philips
92	Female	19.5	10	-	+	-	-	-	-	-	20.6	-	6	-	-	0.000099	Philips
94	Female	20.9	19	-	+	-	+	+	-	-	27.3	-	3	-	-	0.000134	Philips
94	Female	25.6	17	+	-	+	-	-	-	-	19.0	-	4	-	-	0.000070	GE

Severe gait disturbance was defined as TUG >16.7 s (+3s.d. from the mean). a) the sum of periventricular and deep WMH scores, ranging from 0 to 6. HT=Hypertension; DM=Diabetes mellitus; AF=Atrial fibrillation; CAD=Coronary artery disease; HF=Heart failure; + = present; - = absent; na=not available

Table S3: The list of participants with unsuitable MRI for quantitative analysis

age	sex	Reason for omission
66	Male	Right and left temporal lobe contusion
66	Male	Right putamen and corona radiata infarction
66	Male	Left putaminal hemorrhage
66	Female	Frontal lobe infarction
66	Female	Right putaminal infarction
67	Male	Left putaminal infarction
67	Female	Left frontal lobe hemorrhage
69	Female	Metal artefact, post clipping for aneurysm
69	Male	Right temporo-occipital infarction
69	Male	Left frontal lobe infarction
69	Male	Metal artefact (right middle cerebral artery)
69	Male	Right occipital lobe, right putamen and left frontal lobe infarction
70	Male	Right and left temporal lobe infarction
70	Female	Right corona radiata infarction
71	Female	Skull base tumor
71	Female	Right frontal lobe infarction
71	Female	Left cerebellum infarction
72	Male	Left frontal lobe and right occipital lobe infarction
72	Female	Right putamen and corona radiata infarction
72	Male	Meningioma
73	Female	Left frontal lobe contusion
73	Male	Right putaminal hemorrhage
73	Female	Right temporal lobe infarction
74	Female	Metal artefact (post clipping for aneurysm), cerebellum infarction
75	Male	Right parietal lobe infarction
75	Male	Right frontal lobe contusion
75	Female	Metal artefact (right middle cerebral artery)
76	Male	Left frontal lobe infarction
76	Male	Right occipital lobe infarction, right putaminal hemorrhage
77	Female	Left frontal and occipital lobe infarction
77	Female	Left parietal lobe infarction
77	Female	Right putaminal hemorrhage
78	Male	Right putaminal infarction

78	Male	Left putaminal infarction
78	Female	Right frontal and temporal lobe contusion
79	Male	Left occipital lobe infarction
79	Male	Right temporal and parietal lobe infarction
79	Male	Left temporo-occipital infarction
80	Female	Metal artefact
80	Male	Left frontal lobe infarction
80	Male	Right occipital lobe and left frontal lobe infarction
81	Male	Left putaminal infarction
81	Female	Left putaminal hemorrhage
82	Female	Left middle cerebral artery territory infarction
82	Male	Left occipital lobe infarction
82	Female	Right putaminal infarction, Right temporo-occipital infarction
82	Male	Left temporo-occipital infarction
84	Female	Right frontal lobe contusion
85	Female	Right frontal lobe contusion
86	Female	Left subdural hygroma
86	Female	Left frontal lobe contusion and infarction
87	Male	Motion artefact
88	Female	Metal artefact, post clipping for aneurysm
89	Male	Right and left thalamic hemorrhage
90	Male	Left frontal lobe contusion
90	Male	Right and left subdural hygroma
92	Male	Right pons infarction
94	Female	Artefact (shunt tube)

Table S4: Proportion of missing data for variables recorded in this study

Variables	Number of missing data
Hypertension	4 (0.26%)
Diabetes Mellitus	4 (0.26%)
Dyslipidemia	9 (0.60%)
Atrial fibrillation	2 (0.13%)
Heart failure	2 (0.13%)
History of smoking	6 (0.40%)
BMI	2 (0.13%)
MMSE	3 (0.20%)
TUG	8 (0.53%)

**Table S5: The list of brain regions whose volumes were calculated by FreeSurfer**
Frontal lobe
Caudal anterior cingulate
Caudal middle frontal
Lateral orbitofrontal
Medial orbitofrontal
Paracentral
Pars opercularis
Pars orbitalis
Pars triangularis
Precentral
Rostral anterior cingulate
Rostral middle frontal
Superior frontal
Frontal pole
Temporal lobe
Banks sts
Entorhinal
Fusiform
Inferior temporal
Middle temporal
Parahippocampal

Superior temporal
Temporal pole
Transverse temporal
Parietal lobe
Inferior parietal
Isthmus cingulate
Postcentral
Posterior cingulate
Precuneus
Superior parietal
Supramarginal
Occipital lobe
Cuneus
Lateral occipital
Lingual
Pericalcarine
Insula
Subcortical
Accumbens
Amygdala
Caudate
Hippocampus
Putamen

Pallidum
Thalamus
Corpus callosum
Cerebellum
Cerebellum cortex
Vessels

Table S6: Comparison between characteristics of MCI group and cognitively normal group in individuals without DESH

	MCI group (n=213)	Cognitively normal group (n=1,118)	*P*-value
Age	77.6 (6.3)	72.9 (5.8)	< 0.0001*
Female	109 (51.2%)	712 (63.7%)	0.0006*
Education (≤ 9 years)	88 (41.3%)	261 (23.3%)	< 0.0001*
Hypertension	159 (74.6%)	792 (70.8%)	0.2595
Diabetes mellitus	35 (16.4%)	169 (15.1%)	0.6252
Dyslipidemia	98 (46.0%)	585 (52.3%)	0.0910
Atrial fibrillation	19 (8.9%)	60 (5.4%)	0.0443*
Coronary artery disease	9 (4.2%)	57 (5.1%)	0.5906
Heart failure	6 (2.8%)	13 (1.2%)	0.0622
BMI	23.6 (3.5)	23.5 (3.2)	0.7814
History of smoking	47 (22.1%)	302 (27.0%)	0.1325
MMSE	24.6 (2.5)	28.0 (2.0)	< 0.0001*
TUG	9.4 (2.1)	8.4 (1.6)	< 0.0001*
Cognitive impairment [a)]	213 (100%)	0 (0.0%)	< 0.0001*
Gait disturbance [b)]	24 (11.3%)	30 (2.7%)	< 0.0001*
Imaging			
MRI scanner (Philips)	144 (67.6%)	740 (66.2%)	0.6884
Fazekas score [c)]	2.8 (2.3)	2.0 (2.1)	< 0.0001*
Lacunar infarction	65 (30.5%)	222 (19.9%)	0.0005*

Microbleeds	30 (14.1%)	151 (13.5%)	0.8215
Perivascular space	0.0001 (0.00005)	0.0001 (0.00005)	0.1675
VS	0.0372 (0.0097)	0.0323 (0.0079)	< 0.0001*
SF	0.0143 (0.0025)	0.0134 (0.0020)	< 0.0001*
SHM	0.0351 (0.0080)	0.0378 (0.0068)	< 0.0001*

Data are n (%) or mean (SD).

a) MCI.

b) TUG>12.0 s.

c) the sum of periventricular and deep WMH scores, ranging from 0 to 6.

*Significance at level $P<0.05$.

The volume of perivascular space and each DESH-related region were normalised to the total intracranial volume.

Table S7: Association of volume of brain structures with age

	All individuals (n=1,356)		Individuals without DESH (n=1,331)	
	**β_{STD}**	***P*-value**	**β_{STD}**	***P*-value**
Total Brain volume	-0.4161	<0.0001*	-0.4141	<0.0001*
Frontal lobe				
Caudal anterior cingulate	-0.0726	0.0141*	-0.0490	0.1009
Caudal middle frontal	-0.1751	<0.0001*	-0.1720	<0.0001*
Lateral orbitofrontal	-0.2019	<0.0001*	-0.1942	<0.0001*
Medial orbitofrontal	-0.1609	<0.0001*	-0.1499	<0.0001*
Paracentral	-0.2086	<0.0001*	-0.2080	<0.0001*
Pars opercularis	-0.1622	<0.0001*	-0.1619	<0.0001*
Pars orbitalis	-0.1327	<0.0001*	-0.1206	<0.0001*
Pars triangularis	-0.2003	<0.0001*	-0.1942	<0.0001*
Precentral	-0.2021	<0.0001*	-0.2007	<0.0001*
Rostral anterior cingulate	-0.0876	0.0031*	-0.0761	0.0109*
Rostral middle frontal	-0.2535	<0.0001*	-0.2410	<0.0001*
Superior frontal	-0.2694	<0.0001*	-0.2692	<0.0001*
Frontal pole	-0.0118	0.6810	-0.0100	0.7305
Temporal lobe				
Banks sts	-0.2278	<0.0001*	-0.2160	<0.0001*
Entorhinal	-0.2433	<0.0001*	-0.2270	<0.0001*
Fusiform	-0.2666	<0.0001*	-0.2574	<0.0001*
Inferior temporal	-0.2804	<0.0001*	-0.2708	<0.0001*
Middle temporal	-0.2821	<0.0001*	-0.2742	<0.0001*
Parahippocampal	-0.2086	<0.0001*	-0.1942	<0.0001*
Superior temporal	-0.2419	<0.0001*	-0.2315	<0.0001*
Temporal pole	-0.0572	0.0501	-0.0465	0.1152
Transverse temporal	-0.1981	<0.0001*	-0.1911	<0.0001*
Parietal lobe				
Inferior parietal	-0.2552	<0.0001*	-0.2508	<0.0001*
Isthmus cingulate	-0.1447	<0.0001*	-0.1358	<0.0001*
Postcentral	-0.1419	<0.0001*	-0.1434	<0.0001*
Posterior cingulate	-0.1748	<0.0001*	-0.1601	<0.0001*
Precuneus	-0.2057	<0.0001*	-0.2007	<0.0001*
Superior parietal	-0.1934	<0.0001*	-0.1930	<0.0001*
Supramarginal	-0.2115	<0.0001*	-0.2035	<0.0001*
Occipital lobe				
Cuneus	-0.1246	<0.0001*	-0.1264	<0.0001*
Lateral occipital	-0.2711	<0.0001*	-0.2714	<0.0001*
Lingual	-0.2201	<0.0001*	-0.2170	<0.0001*
Pericalcarine	-0.0492	0.1004	-0.0513	0.0897
Insula	-0.1042	0.0001*	-0.0999	0.0002*
Subcortical				
Accumbens	-0.2380	<0.0001*	-0.2405	<0.0001*
Amygdala	-0.3285	<0.0001*	-0.3253	<0.0001*
Caudate	0.0140	0.5985	0.0053	0.8426
Hippocampus	-0.4113	<0.0001*	-0.4062	<0.0001*
Putamen	-0.0767	0.0065*	-0.0801	0.0045*
Pallidum	-0.0559	0.0483	-0.0533	0.0605
Thalamus	-0.2683	<0.0001*	-0.2662	<0.0001*
Corpus callosum	-0.2599	<0.0001*	-0.2519	<0.0001*
Cerebellum				
Cerebellum cortex	-0.2211	<0.0001*	-0.2185	<0.0001*

In the multiple regressions, adjustments were made for sex; education; hypertension; diabetes mellitus; dyslipidaemia;

atrial fibrillation; coronary artery disease; heart failure; body mass index; history of smoking; MRI scanner; Fazekas score; lacunar infarction, microbleeds and perivascular space.

β_{STD}: standardized regression coefficient.

*The significance level was *P*<0.05.

Table S8: Results of hierarchical multiple regression analysis for MMSE with brain structures

	All individuals (n=1,356)						Individuals without DESH (n=1,331)				
	Brain structure	β_{STD}	*P*-value	ΔR^2	$\Delta R^2/R^2$		Brain structure	β_{STD}	*P*-value	ΔR^2	$\Delta R^2/R^2$
1	SHM	0.0993	0.0002*	0.0090	0.0575	1	SHM	0.0910	0.0006*	0.0076	0.0525
2	VS	-0.0916	0.0010*	0.0068	0.0435	2	VS	-0.0932	0.0009*	0.0071	0.0494
3	Accumbens	0.0919	0.0016	0.0062	0.0400	3	Accumbens	0.0914	0.0019	0.0062	0.0429
4	Hippocampus	0.0904	0.0029	0.0056	0.0357	4	Middle temporal	0.0901	0.0020	0.0061	0.0424
5	Middle temporal	0.0852	0.0033	0.0054	0.0348	5	Precuneus	0.0926	0.0024	0.0059	0.0409
6	SF	-0.0783	0.0042	0.0052	0.0331	6	Hippocampus	0.0926	0.0024	0.0059	0.0409
7	Precuneus	0.0863	0.0043	0.0051	0.0329	7	Lateral occipital	0.0807	0.0051	0.0050	0.0350
8	Lateral occipital	0.0808	0.0047	0.0050	0.0322	8	Amygdala	0.0819	0.0051	0.0050	0.0349
9	Supramarginal	0.0799	0.0052	0.0049	0.0315	9	Banks sts	0.0822	0.0053	0.0050	0.0346
10	Amygdala	0.0811	0.0052	0.0049	0.0315	10	Supramarginal	0.0797	0.0056	0.0049	0.0342
11	Lingual	0.0708	0.0107	0.0041	0.0263	11	Lingual	0.0725	0.0096	0.0043	0.0299
12	Transverse temporal	0.0685	0.0125	0.0039	0.0252	12	SF	-0.0704	0.0104	0.0042	0.0292
13	Superior temporal	0.0704	0.0161	0.0036	0.0234	13	Superior temporal	0.0724	0.0141	0.0039	0.0269
14	Lateral orbitofrontal	0.0658	0.0196	0.0034	0.0220	14	Superior frontal	0.0756	0.0143	0.0039	0.0267
15	Superior frontal	0.0712	0.0200	0.0034	0.0219	15	Inferior parietal	0.0714	0.0188	0.0035	0.0246
16	Inferior parietal	0.0675	0.0254	0.0031	0.0202	16	Inferior temporal	0.0649	0.0220	0.0034	0.0234
17	Banks sts	0.0647	0.0263	0.0031	0.0199	17	Transverse temporal	0.0632	0.0225	0.0033	0.0232
18	Inferior temporal	0.0595	0.0347	0.0028	0.0180	18	Lateral orbitofrontal	0.0641	0.0236	0.0033	0.0228
19	Precentral	0.0711	0.0367	0.0028	0.0176	19	Precentral	0.0630	0.0669	0.0022	0.0150
20	Entorhinal	0.0487	0.0659	0.0021	0.0137	20	Entorhinal	0.0469	0.0784	0.0020	0.0138
21	Fusiform	0.0494	0.0786	0.0020	0.0125	21	Fusiform	0.0493	0.0811	0.0020	0.0136
22	Pars orbitalis	0.0461	0.0908	0.0018	0.0116	22	Insula	0.0489	0.0977	0.0018	0.0122
23	Thalamus	0.0466	0.1046	0.0017	0.0106	23	Pars orbitalis	0.0452	0.0999	0.0017	0.0121
24	Pars opercularis	0.0426	0.1149	0.0016	0.0100	24	Pars triangularis	0.0403	0.1357	0.0014	0.0099
25	Insula	0.0459	0.1160	0.0016	0.0100	25	Pars opercularis	0.0394	0.1488	0.0013	0.0093
26	Pars triangularis	0.0397	0.1385	0.0014	0.0089	26	Posterior cingulate	0.0373	0.1901	0.0011	0.0077
27	Posterior cingulate	0.0371	0.1901	0.0011	0.0069	27	Thalamus	0.0363	0.2082	0.0010	0.0071
28	Pallidum	0.0354	0.2000	0.0010	0.0066	28	Pallidum	0.0313	0.2645	0.0008	0.0056
29	Parahippocampal	0.0342	0.2267	0.0009	0.0059	29	Parahippocampal	0.0282	0.3228	0.0006	0.0044
30	Postcentral	0.0307	0.2891	0.0007	0.0045	30	Postcentral	0.0272	0.3524	0.0006	0.0039
31	Putamen	0.0255	0.3583	0.0005	0.0034	31	Cuneus	0.0247	0.3563	0.0005	0.0038
32	Cuneus	0.0212	0.4244	0.0004	0.0026	32	Rostral anterior cingulate	0.0243	0.3628	0.0005	0.0037
33	Superior parietal	0.0243	0.4293	0.0004	0.0025	33	Caudal middle frontal	0.0242	0.3918	0.0005	0.0033
34	Caudal middle frontal	0.0209	0.4545	0.0004	0.0023	34	Rostral middle frontal	0.0225	0.4152	0.0004	0.0030
35	Rostral middle frontal	0.0202	0.4636	0.0003	0.0022	35	Superior parietal	0.0250	0.4211	0.0004	0.0029
36	Rostral anterior cingulate	0.0189	0.4749	0.0003	0.0021	36	Cerebellum cortex	-0.0201	0.4546	0.0004	0.0025
37	Caudate	0.0204	0.4880	0.0003	0.0019	37	Putamen	0.0195	0.4902	0.0003	0.0021
38	Isthmus cingulate	0.0169	0.5350	0.0002	0.0016	38	Caudate	0.0195	0.5104	0.0003	0.0019
39	Cerebellum cortex	-0.0132	0.6206	0.0002	0.0010	39	Isthmus cingulate	0.0151	0.5812	0.0002	0.0014
40	Pericalcarine	0.0112	0.6683	0.0001	0.0007	40	Pericalcarine	0.0143	0.5875	0.0002	0.0013
41	Frontal pole	0.0076	0.7781	0.0001	0.0003	41	Temporal pole	0.0069	0.7976	0.0000	0.0003
42	Corpus callosum	0.0079	0.7888	0.0000	0.0003	42	Medial orbitofrontal	0.0067	0.8004	0.0000	0.0003
43	Medial orbitofrontal	0.0040	0.8782	0.0000	0.0001	43	Caudal anterior cingulate	-0.0050	0.8526	0.0000	0.0002
44	Paracentral	-0.0039	0.8940	0.0000	0.0001	44	Frontal pole	0.0046	0.8659	0.0000	0.0001
45	Temporal pole	0.0020	0.9416	0.0000	0.0000	45	Paracentral	0.0021	0.9426	0.0000	0.0000
46	Caudal anterior cingulate	-0.0019	0.9428	0.0000	0.0000	46	Corpus callosum	0.0005	0.9866	0.0000	0.0000

After controlling for age, sex, education, hypertension, diabetes mellitus, dyslipidaemia, atrial fibrillation, coronary artery disease, heart failure, body mass index, history of smoking, MRI scanner, Fazekas score, lacunar infarction, microbleeds and perivascular space (Block 1), each brain structure was entered in Block 2. Forty-six brain structures in descending order of ΔR^2 are shown. β_{STD}: standardized regression coefficient. *The significance level was P<0.0011 to correct for 46 modeling analyses.

Table S9: Results of hierarchical multiple regression analysis for TUG with brain structures

All individuals (n=1,356)						Individuals without DESH (n=1,331)					
	Brain structure	β_{STD}	*P*-value	ΔR^2	$\Delta R^2/R^2$		Brain structure	β_{STD}	*P*-value	ΔR^2	$\Delta R^2/R^2$
1	Precentral	-0.1534	<0.0001*	0.0128	0.0876	1	Precentral	-0.1433	<0.0001*	0.0112	0.0799
2	Pars orbitalis	-0.1188	<0.0001*	0.0120	0.0818	2	Pars orbitalis	-0.1091	<0.0001*	0.0101	0.0725
3	Lateral orbitofrontal	-0.1227	<0.0001*	0.0119	0.0817	3	Lateral orbitofrontal	-0.1115	<0.0001*	0.0100	0.0712
4	Lingual	-0.1127	<0.0001*	0.0104	0.0711	4	Lingual	-0.1078	0.0001*	0.0095	0.0682
5	Accumbens	-0.1163	<0.0001*	0.0100	0.0684	5	Superior parietal	-0.1148	0.0002*	0.0088	0.0630
6	Superior parietal	-0.1216	<0.0001*	0.0099	0.0676	6	Accumbens	-0.1073	0.0003*	0.0085	0.0610
7	Insula	-0.1123	0.0001*	0.0093	0.0637	7	Insula	-0.1013	0.0006*	0.0076	0.0541
8	Superior frontal	-0.1134	0.0002*	0.0086	0.0591	8	Precuneus	-0.1040	0.0007*	0.0074	0.0533
9	Superior temporal	-0.1082	0.0002*	0.0086	0.0589	9	Superior frontal	-0.1050	0.0007*	0.0074	0.0532
10	Supramarginal	-0.1049	0.0002*	0.0085	0.0581	10	Middle temporal	-0.0983	0.0008*	0.0073	0.0522
11	Posterior cingulate	-0.1032	0.0003*	0.0084	0.0574	11	Superior temporal	-0.0970	0.0010*	0.0070	0.0498
12	Middle temporal	-0.1056	0.0003*	0.0083	0.0570	12	Supramarginal	-0.0934	0.0012	0.0068	0.0484
13	Precuneus	-0.1075	0.0004*	0.0080	0.0544	13	Inferior temporal	-0.0918	0.0012	0.0067	0.0482
14	Pallidum	-0.0958	0.0005*	0.0076	0.0518	14	Pallidum	-0.0878	0.0018	0.0063	0.0451
15	Hippocampus	-0.1005	0.0009*	0.0069	0.0471	15	Hippocampus	-0.0955	0.0018	0.0063	0.0450
16	Inferior temporal	-0.0923	0.0010*	0.0068	0.0462	16	Posterior cingulate	-0.0851	0.0028	0.0058	0.0412
17	Transverse temporal	-0.0892	0.0011	0.0067	0.0455	17	Corpus callosum	-0.0857	0.0039	0.0054	0.0386
18	Corpus callosum	-0.0900	0.0022	0.0059	0.0404	18	Banks sts	-0.0850	0.0040	0.0053	0.0382
19	Banks sts	-0.0882	0.0024	0.0058	0.0396	19	Caudate	-0.0822	0.0056	0.0050	0.0354
20	Rostral anterior cingulate	-0.0790	0.0028	0.0056	0.0385	20	Transverse temporal	-0.0736	0.0080	0.0045	0.0325
21	Medial orbitofrontal	-0.0752	0.0043	0.0051	0.0350	21	Rostral anterior cingulate	-0.0695	0.0094	0.0044	0.0312
22	Parahippocampal	-0.0796	0.0048	0.0050	0.0341	22	Parahippocampal	-0.0731	0.0105	0.0042	0.0303
23	Pars opercularis	-0.0756	0.0051	0.0049	0.0338	23	Amygdala	-0.0709	0.0156	0.0038	0.0271
24	Caudal anterior cingulate	-0.0733	0.0055	0.0048	0.0331	24	Inferior parietal	-0.0733	0.0161	0.0037	0.0268
25	VS	0.0763	0.0062	0.0047	0.0323	25	Pars opercularis	-0.0653	0.0167	0.0037	0.0265
26	Rostral middle frontal	-0.0748	0.0064	0.0047	0.0320	26	Temporal pole	-0.0645	0.0173	0.0037	0.0262
27	Fusiform	-0.0763	0.0065	0.0047	0.0319	27	Fusiform	-0.0659	0.0200	0.0035	0.0250
28	Caudate	-0.0773	0.0084	0.0044	0.0299	28	Caudal anterior cingulate	-0.0620	0.0204	0.0035	0.0249
29	Temporal pole	-0.0702	0.0086	0.0043	0.0297	29	Medial orbitofrontal	-0.0613	0.0212	0.0034	0.0245
30	Inferior parietal	-0.0781	0.0096	0.0042	0.0289	30	Cuneus	-0.0611	0.0228	0.0033	0.0240
31	Amygdala	-0.0743	0.0105	0.0041	0.0282	31	Cerebellum cortex	-0.0598	0.0264	0.0032	0.0228
32	Cuneus	-0.0678	0.0105	0.0041	0.0282	32	Rostral middle frontal	-0.0607	0.0284	0.0031	0.0222
33	Lateral occipital	-0.0727	0.0110	0.0041	0.0278	33	Postcentral	-0.0632	0.0311	0.0030	0.0215
34	Cerebellum cortex	-0.0666	0.0124	0.0039	0.0269	34	Lateral occipital	-0.0609	0.0350	0.0029	0.0206
35	Postcentral	-0.0719	0.0129	0.0039	0.0266	35	Putamen	-0.0579	0.0410	0.0027	0.0193
36	Putamen	-0.0661	0.0170	0.0036	0.0245	36	Paracentral	-0.0572	0.0514	0.0025	0.0176
37	Isthmus cingulate	-0.0624	0.0219	0.0033	0.0226	37	VS	0.0533	0.0579	0.0023	0.0166
38	Entorhinal	-0.0606	0.0222	0.0033	0.0225	38	Caudal middle frontal	-0.0534	0.0594	0.0023	0.0164
39	Frontal pole	-0.0607	0.0251	0.0032	0.0216	39	Frontal pole	-0.0509	0.0634	0.0022	0.0159
40	Caudal middle frontal	-0.0543	0.0522	0.0024	0.0162	40	Isthmus cingulate	-0.0499	0.0695	0.0021	0.0152
41	Pars triangularis	-0.0473	0.0779	0.0020	0.0134	41	Entorhinal	-0.0462	0.0838	0.0019	0.0138
42	Paracentral	-0.0504	0.0809	0.0019	0.0131	42	Pars triangularis	-0.0389	0.1505	0.0013	0.0096
43	SHM	-0.0441	0.0939	0.0018	0.0121	43	Thalamus	-0.0403	0.1639	0.0013	0.0090
44	Pericalcarine	-0.0416	0.1107	0.0016	0.0110	44	Pericalcarine	-0.0274	0.3004	0.0007	0.0050
45	Thalamus	-0.0458	0.1112	0.0016	0.0109	45	SF	0.0258	0.3493	0.0006	0.0041
46	SF	0.0394	0.1495	0.0013	0.0090	46	SHM	-0.0171	0.5192	0.0003	0.0019

After controlling for age, sex, education, hypertension, diabetes mellitus, dyslipidaemia, atrial fibrillation, coronary artery disease, heart failure, body mass index, history of smoking, MRI scanner, Fazekas score, lacunar infarction, microbleeds and perivascular space (Block 1), each brain structure was entered in Block 2. Forty-six brain structures in descending order of ΔR^2 are shown. β_{STD}: standardized regression coefficient. *The significance level was P<0.0011 to correct for 46 modeling analyses.
